# Flexible network reconstruction from relational databases with Cytoscape and CytoSQL

**DOI:** 10.1186/1471-2105-11-360

**Published:** 2010-07-01

**Authors:** Kris Laukens, Jens Hollunder, Thanh Hai Dang, Geert De Jaeger, Martin Kuiper, Erwin Witters, Alain Verschoren, Koenraad Van Leemput

**Affiliations:** 1 Department of Mathematics and Computer Science, University of Antwerp, Middelheimlaan 1, B-2020 Antwerpen, Belgium; 2 Department of Plant Systems Biology, Flanders Institute for Biotechnology and Department of Molecular Genetics, Ghent University, Technologiepark 927, 9052 Ghent, Belgium; 3 Department of Biology, NTNU, Høgskoleringen 5, N-7491 Trondheim, Norway; 4 Flemish Institute for Technological Research (VITO), Boeretang 200, B-2400 Mol, Belgium; 5 Department of Biology, University of Antwerp, Groenenborgerlaan 171, B-2020 Antwerpen, Belgium

## Abstract

**Background:**

Molecular interaction networks can be efficiently studied using network visualization software such as Cytoscape. The relevant nodes, edges and their attributes can be imported in Cytoscape in various file formats, or directly from external databases through specialized third party plugins. However, molecular data are often stored in relational databases with their own specific structure, for which dedicated plugins do not exist. Therefore, a more generic solution is presented.

**Results:**

A new Cytoscape plugin 'CytoSQL' is developed to connect Cytoscape to any relational database. It allows to launch SQL ('Structured Query Language') queries from within Cytoscape, with the option to inject node or edge features of an existing network as SQL arguments, and to convert the retrieved data to Cytoscape network components. Supported by a set of case studies we demonstrate the flexibility and the power of the CytoSQL plugin in converting specific data subsets into meaningful network representations.

**Conclusions:**

CytoSQL offers a unified approach to let Cytoscape interact with relational databases. Thanks to the power of the SQL syntax, this tool can rapidly generate and enrich networks according to very complex criteria. The plugin is available at http://www.ptools.ua.ac.be/CytoSQL.

## Background

Molecular interactions are the focus of many functional genomics studies, and they form a cornerstone of Systems Biology research. Software to integrate and analyse these interactions and their attributes plays an increasingly important role. The most widely used open source network visualization workbench is Cytoscape [1]. Data from various sources can be imported into this tool to build networks, and to highlight specific node or edge features. Cytoscape can also be used to filter and browse a network and it offers extensive data mining possibilities. Many of these features are provided or extended through third-party plugins.

The potential of Cytoscape to integrate heterogeneous data relies heavily on its data import capabilities. Import functions for several common file types and connectivity to web services are built in. A number of third party plugins, such as BioNetBuilder [2], the MiSink plugin [3], BiNOM [4] and MiMI [5] provide a dedicated interface between Cytoscape and specific data sources. Nevertheless, the standard import capabilities and the existing range of import plugins have certain limitations. Interactions and their attributes are widely collected into relational databases, with their own specific structure and maintained by different institutes and organisations. In bioinformatics facilities that support wet laboratories, experiments are often stored in local, internally developed and continuously changing databases. Usually such databases are not supported by an existing Cytoscape plugin. Exporting data from these databases as text and importing it into Cytoscape is possible, but takes time, and needs to be repeated every time the network needs to be updated with the most current database information. This process becomes even more cumbersome if a user wants to import new attributes from a database for all given nodes or edges of the network. One way to achieve this consists of exporting the full attribute table from the database, and let Cytoscape filter out the attributes relevant for the given network. This is possible, as long as the attribute table is small enough. An alternative approach would be to export all node (or edge) identifiers from the network, and to query the attribute table of the database with these identifiers. This repeated process of exporting from Cytoscape, querying the database and importing the results back in Cytoscape is particularly time consuming.

To tackle these issues, we propose a unified solution that works with virtually any accessible relational database. The approach was implemented as a Cytoscape plugin called 'CytoSQL', which connects Cytoscape directly with any relational database through a generic method. With this plugin, users can construct and extend networks using "Structured Query Language" (SQL) syntax, a well known database interaction language.

## Implementation

CytoSQL was implemented in Java as a Cytoscape plugin. It interfaces with relational databases through the JDBC driver, which offers an abstraction of most relational database systems. The required drivers for MySQL, SQLite and PostgreSQL are included in the default installation, and the online documentation explains how to configure the plugin for use with alternative drivers or drivers for other database systems. The plugin website http://www.ptools.ua.ac.be/CytoSQL also offers an example relational (MySQL) database structure, along with a tutorial and a number of Python scripts to populate the example database with publicly available test case data.

### Installation and testing

All local database instances were running on an Ubuntu 6.06 LTS server running MySQL 5.0.22. CytoSQL and the application cases were tested with Cytoscape version 2.6.3 and earlier, on Microsoft Windows XP, Ubuntu 8.04.1 and Mac OS × 10.5.8.

The CytoSQL plugin can be installed from the Cytoscape plugin manager, under the "Network and Attribute I/O" category.

### User interface

After installation, CytoSQL can be launched from the plugin menu and it offers access to the query window and to the database definition window.

#### Database definition

**Figure 1:**
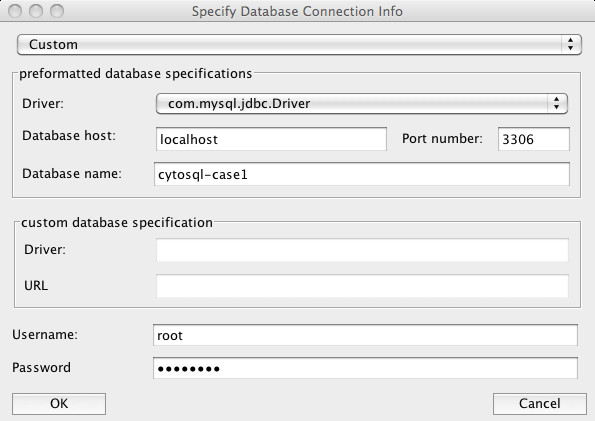
**Screenshot of the CytoSQL database connectivity window**. The database connectivity window allows to define and store database connection settings and enables rapid switching between different databases.

Prior to any query execution, its connection must be defined. The database definition window (Figure 1) collects common database access coordinates. The form supports storage and retrieval of multiple connection settings and facilitates quick switching between different databases. It is thus straightforward to extract information from different databases in one single session.

#### Query window

**Figure 2:**
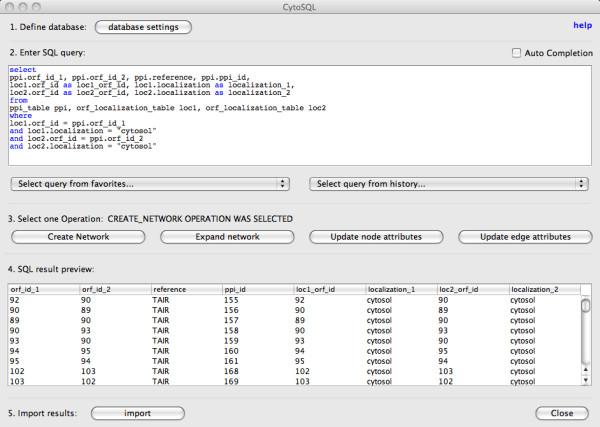
**Screenshot of the CytoSQL query window**. The main CytoSQL window allows to define, save, evaluate and launch queries.

The main CytoSQL window (Figure 2) lets the user specify, test and launch SQL statements against the selected database. In the query definition field the user can enter any SQL 'SELECT' statement. The recent query history is available through a drop-down box that allows to rapidly relaunch a previous query. A second drop-down box enables users to store and launch a set of favorite queries, which can be edited and managed in a dedicated editor window. The query can then be executed through one of the four modes of operation: create network, expand network, update node attributes or update edge attributes. Depending on the chosen mode of operation, a query may support 'bind variables'. Bind variables are 'replacement' variables that a user can add into the SQL query. They are instantiated with chosen attributes of currently selected Cytoscape network nodes or edges. A preview field enables the user to verify the results of the query, prior to finalizing the import of the results as network elements into Cytoscape.

## Results

### Launching queries and mapping results

After execution of the query, the resulting rows and columns are subsequently mapped to Cytoscape network elements in a way determined by the selected mode of operation:

#### Mode 1: Creating a new network

The first and most simple mode is the creation of a new Cytoscape network through one 'SELECT' statement. Result sets of SQL statements are returned in single table format, consisting of rows (records) and columns. The plugin interface prompts the user to specify for each column whether it concerns the source node, the target node or an attribute of the source, target or edge (see documentation for more details). The Cytoscape network is immediately generated hereafter. Field types (integer, double, string, ...) are automatically converted to equivalent Cytoscape data types.

For example:

SELECT protn1, protn2, intType, confirmed FROM interactions WHERE confirmed = 'Y'

This hypothetical query could retrieve two interacting nodes (protn1 and protn2), and two edge attributes for this interaction (interaction type "intType" and whether the interaction is confirmed or not).

#### Mode 2: Loading attributes for selected nodes

A second mode of operation enables loading new attributes for a set of selected nodes or - if no nodes are selected - for all nodes in the network. The user can put one or more 'bind' (replacement) variables (represented by question marks) in the 'WHERE' clause of the SQL query. A plugin dialog box then asks the user which Cytoscape node field(s) should replace the bind variable(s). The query will be subsequently launched for each node individually with the appropriate variable(s) replaced by the value(s) of the selected node fields.

For example:

SELECT protein, mass, pI FROM features WHERE protein = ?

In this example, the Cytoscape node identifier may be mapped to the bind variable "?", and the query is launched once for each node, retrieving "mass" and pI" as new node attributes.

#### Mode 3: Loading attributes for selected edges

In a fashion similar to Mode 2, the plugin can load new attributes for existing edges, based on one or more replacement variables in the 'WHERE' clause. Both node or edge attribute fields can be used to replace this variable, and the query will be launched once for each edge.

For example:

SELECT protn1, protn2, intType, score FROM predictedInteractions WHERE protn1 = ? AND protn2 = ?

The query contains two bind variables, each of which is populated with a different Cytoscape attribute, in this hypothetical example the interacting node identifiers of each selected edge. The query loads predicted information (type, score) for interactions that are already present in the network.

#### Mode 4: Expanding an existing network

The last mode of operation is used to extend an existing network, by loading new nodes that interact with the selected nodes, together with their edges. This mode also requires the use of at least one bind variable, that inserts a Cytoscape node attribute in the 'WHERE' clause of the SQL query.

For example:

SELECT protn2, intType, score FROM predictedInteractions WHERE protn1 = ?

This query loads new interactions that originate from selected nodes.

A toy example database against which aforementioned example queries can be executed, is provided on the plugin website.

### Application cases

To demonstrate the functionalities of the CytoSQL plugin with real data and to facilitate its comparison with alternative import options, we describe its use in a series of application cases.

#### Case 1: Rapid reconstruction of complex networks

To demonstrate how complex networks can be rapidly reconstructed from relational databases, we installed a local instance of the UCSC (University of California Santa Cruz) proteome browser MySQL database [6]. Using a single SQL statement, combining three different tables, we built a yeast-specific network (Additional file 1) consisting of proteins and domains as two different types of nodes, and in which edges between both node types represent the fact that a given protein contains a given domain. The graph offers a global view on the distribution of protein-domain memberships and could, in combination with protein interaction data, for example serve as a starting point to study the importance of domains in protein interactions. The relative simplicity of this query highlights the power of SQL statements in extracting specific subsets from large databases: individually loading these complete three tables in Cytoscape could easily reach internal memory constraints.

#### Case 2: Enrichment of existing networks

In the next case study, we demonstrate the enrichment functions of the CytoSQL plugin, on a reaction network from a local instance of the Reactome database [7-9]. The first two queries are used to generate a network consisting of relationships between "molecules" and the "reactions" between them. To demonstrate the simplicity of using CytoSQL to enrich an existing network, we added useful node attributes for reactions and molecules, with one extra query, which loads molecule and reaction descriptions for selected (or all) nodes. The resulting network view and detailed queries are shown in Additional file 2. Executing the same task through file export and import functions requires exporting the full (and potentially large) attribute table from the database, and importing it into Cytoscape. Using CytoSQL, only the attributes for relevant nodes need to be loaded and this is done in one single step.

#### Case 3: Use of public, remote relational databases

CytoSQL is also useful for the local integration of data from remote databases. As a demonstration, we employed 'BioWarehouse', a Bioinformatics toolkit to collect biological data from multiple databases into a so-called "data warehouse" [10]. A public instance is available for users (after free registration). Using a single query, we constructed a network representation of the relationships between different "terms" in the warehouse. The network representation (Additional file 3) visualizes the structure of the relationships between terms, and reveals how terms belonging to different ontologies group together, and whether and how terms within an ontology are hierarchically organized. Other warehouse systems exist that offer similar possibilities to query heterogeneous and complex data with SQL statements [11-13].

## Discussion

Network reconstruction has become a major aspect in computational analysis of biological data. This task is often dependent on data distributed in distinct databases. Correct combinatorial analysis of data residing in different sources usually requires downloading and combining each dataset, a task that may become challenging if some of these datasets are large, whereas the intersect subset that is actually required may still be small. Relational databases facilitate the task of combining data and generating subsets of data based on complex criteria. The tool presented here unifies all types of interactions with databases into a single Cytoscape plugin. Due to the query language and the generic nature of the plugin, it facilitates otherwise complex data import and export procedures and it offers a valuable complement to existing Cytoscape plugins, which are often focussed on certain data sources or on very specific applications.

This ability to import data from any user accessible database simplifies otherwise extensive processing tasks for network generation, network analysis, and network attribute manipulation, to one or more SQL statements. Since SQL syntax supports data conversion through string and numeric functions, even small data manipulations can be accomplished during import. The bind variable option allows the user to incrementally extend or enrich an existing network, through any combination of node and edge attributes.  Together with the easy connection switching option a user can sequentially interconnect independent relational databases using selected attributes, allowing the user to gradually integrate various data sources while generating visual network representations.

The plugin may be utilized to connect to local databases, but can also be used to connect to any relational database that the user has sufficient access permissions for. Potential disadvantages of this approach are the requirement of unwrapped SQL connections and the related security risks. For internal applications this does not pose problems, but externally only a limited number of databases are directly accessible by SQL statements. The replication or "mirroring" of data repositories may mitigate this problem and bring the full query potential within any lab. With its generic approach, this plugin complements existing plugins, but is does not replace the available plugins that are specifically designed to offer biologist access to common databases. A relevant drawback of this plugin is the fact that a user is assumed to have some knowledge of SQL. It is also essential that the user understands the structure of the database to be queried. These factors may be a disadvantage for biologists and make the tool primarily relevant for bioinformaticians. Nevertheless, with a basic understanding of the 'SELECT' statement syntax however, the user can already accomplish most network generation tasks. Right now the plugin supports storing queries. Future developments may include a graphical query builder and tools to exchange stored queries among users, in order to bring its power within reach of a larger user base.

## Conclusions

Relationships between biological data become increasingly relevant and are more and more studied with high throughput techniques. It can be expected that the amount of available relational information will grow rapidly the coming years. This plugin leverages the use of omnipresent relational data through offering generic access to this information.

### Availability and requirements

- Project name: CytoSQL

- Project home page: http://www.ptools.ua.ac.be/CytoSQL

- Operating system(s): Platform independent

- Programming language: Java

- Other requirements: Cytoscape 2.6.1 or higher

- License: GNU LGPL3

- Any restrictions to use by non-academics: none

## Authors' contributions

KL, DTH, KVL and JH developed and tested the application, KL wrote the manuscript, GDJ, MK and EW were involved in drafting the initial concepts and provided real application cases, and AV coordinated the project. All authors read and approved the final manuscript.

## Supplementary Material

Additional file 1 **AdditionalFile1.pdf - Application case 1**. A pdf document that lists the queries and shows the generated network of application case 1: Rapid reconstruction of complex networks. Click here for file

Additional file 2 **AdditionalFile2.pdf - Application case 2**. A pdf document that lists the queries and shows the generated network of application case 2: Enrichment of existing networks. Click here for file

Additional file 3 **AdditionalFile3.pdf - Application case 3**. A pdf document that lists the queries and shows the generated network of application case 3: Use of public, remote relational databases. Click here for file

## References

[B1] Shannon P Markiel A Ozier O Baliga NS Wang JT Ramage D Amin N Schwikowski B Ideker T Cytoscape: a software environment for integrated models of biomolecular interaction networks Genome Research 2003 13 11 2498 2504 10.1101/gr.1239303 14597658 PMC403769

[B2] Avila-Campillo I Drew K Lin J Reiss DJ Bonneau R BioNetBuilder: automatic integration of biological networks Bioinformatics 2007 23 3 392 393 10.1093/bioinformatics/btl604 17138585

[B3] Salwinski L Eisenberg D The MiSink Plugin: Cytoscape as a graphical interface to the Database of Interacting Proteins Bioinformatics 2007 23 16 2193 2195 10.1093/bioinformatics/btm304 17553858

[B4] Zinovyev A Viara E Calzone L Barillot E BiNoM: a Cytoscape plugin for manipulating and analyzing biological networks Bioinformatics 2008 24 6 876 877 10.1093/bioinformatics/btm553 18024474

[B5] Jayapandian M Chapman A Tarcea VG Yu C Elkiss A Ianni A Liu B Nandi A Santos C Andrews P Athey B States D Jagadish HV Michigan Molecular Interactions (MiMI): putting the jigsaw puzzle together Nucleic Acids Research 2007 35 Database D566 71 10.1093/nar/gkl859 17130145 PMC1716720

[B6] Hsu F Pringle TH Kuhn RM Karolchik D Diekhans M Haussler D Kent WJ The UCSC Proteome Browser Nucl Acids Res 2005 33 suppl_1 D454 458 15608236 10.1093/nar/gki100 PMC540054

[B7] Joshi-Tope G Gillespie M Vastrik I D'Eustachio P Schmidt E de Bono B Jassal B Gopinath G Wu G Matthews L Lewis S Birney E Stein L Reactome: a knowledgebase of biological pathways Nucl Acids Res 2005 33 suppl_1 D428 432 15608231 10.1093/nar/gki072 PMC540026

[B8] Vastrik I D'Eustachio P Schmidt E Joshi-Tope G Gopinath G Croft D de Bono B Gillespie M Jassal B Lewis S Matthews L Wu G Birney E Stein L Reactome: a knowledge base of biologic pathways and processes Genome Biology 2007 8 3 R39 10.1186/gb-2007-8-3-r39 17367534 PMC1868929

[B9] Matthews L Gopinath G Gillespie M Caudy M Croft D de Bono B Garapati P Hemish J Hermjakob H Jassal B Kanapin A Lewis S Mahajan S May B Schmidt E Vastrik I Wu G Birney E Stein L D'Eustachio P Reactome knowledgebase of human biological pathways and processes Nucleic Acids Research 2009 37 Database D619 622 10.1093/nar/gkn863 18981052 PMC2686536

[B10] Lee TJ Pouliot Y Wagner V Gupta P Stringer-Calvert DWJ Tenenbaum JD Karp PD BioWarehouse: a bioinformatics database warehouse toolkit BMC Bioinformatics 2006 7 170 10.1186/1471-2105-7-170 16556315 PMC1444936

[B11] Shah SP Huang Y Xu T Yuen MMS Ling J Ouellette BFF Atlas - a data warehouse for integrative bioinformatics BMC Bioinformatics 2005 6 34 10.1186/1471-2105-6-34 15723693 PMC554782

[B12] Hart RK Mukhyala K Unison: an integrated platform for computational biology discovery Pacific Symposium on Biocomputing 2009 403 414 19209718

[B13] Toepel T Kormeier B Klassen A Hofestaedt R BioDWH: A data warehouse kit for life science data integration Journal of Integrative Bioinformatics 2008 5 2 93 10.2390/biecoll-jib-2008-93 20134070

