# Supplementary material for: Flexible network reconstruction from relational databases with Cytoscape and CytoSQL
Source: BMC Bioinformatics. 2010 Jul 1;11:360. doi: 10.1186/1471-2105-11-360 (PMC2910028; doi:10.1186/1471-2105-11-360)
Supplement: Additional file 1 — AdditionalFile1.pdf - Application case 1. A pdf document that lists the queries and shows the generated network of application case 1: Rapid reconstruction of complex networks. [file 1471-2105-11-360-S1.PDF]

Application 1: UCSC proteome browser

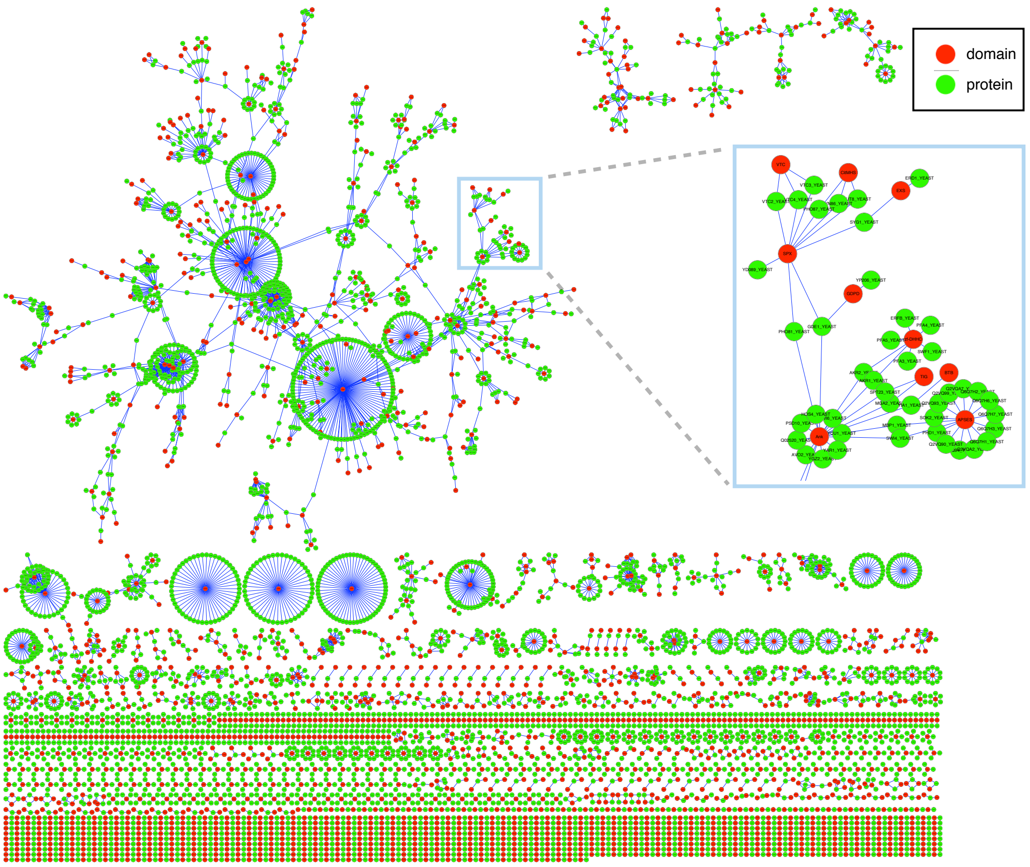

Figure: *Network visualization of Yeast-specific protein-domain membership graph, generated with a single query over three tables of a local instance of the UCSC Proteome browser relational protein database. The rectangle represents a zoomed in view on a small section of the network. A local instance of the UCSC (University of California Santa Cruz, [6]) proteome browser MySQL database (proteinDB - version 080707) was installed. Using a single SQL statement (see Query 1 below), combining three different tables, a yeast-specific network was generated that represents protein-domain memberships as a bipartite graph in which edges represent the fact that a given protein contains a given domain.*

Query 1: Loading a protein-domain membership graph for yeast

```
SELECT
      pfamDesc.pfamAC, pfamXref.swissAC,
      pfamDesc.pfamID, pfamDesc.description,
      pfamXref.pfamAC, pfamXref.swissDisplayID,
      spOrganism.displayID, spOrganism.organism
FROM
      pfamXref, pfamDesc, spOrganism
WHERE
      pfamXref.pfamAC=pfamDesc.pfamAC
AND
      pfamXref.swissDisplayID= spOrganism.displayID
AND
      spOrganism.organism=4932
```

<u>CytoSQL Mode</u>: Create Network
<u>CytoSQL mappings</u>: source node, target node, source, source, source, target, target, target
