# Supplementary material for: Flexible network reconstruction from relational databases with Cytoscape and CytoSQL
Source: BMC Bioinformatics. 2010 Jul 1;11:360. doi: 10.1186/1471-2105-11-360 (PMC2910028; doi:10.1186/1471-2105-11-360)
Supplement: Additional file 2 — AdditionalFile2.pdf - Application case 2. A pdf document that lists the queries and shows the generated network of application case 2: Enrichment of existing networks. [file 1471-2105-11-360-S2.PDF]

Application 2: Reactome

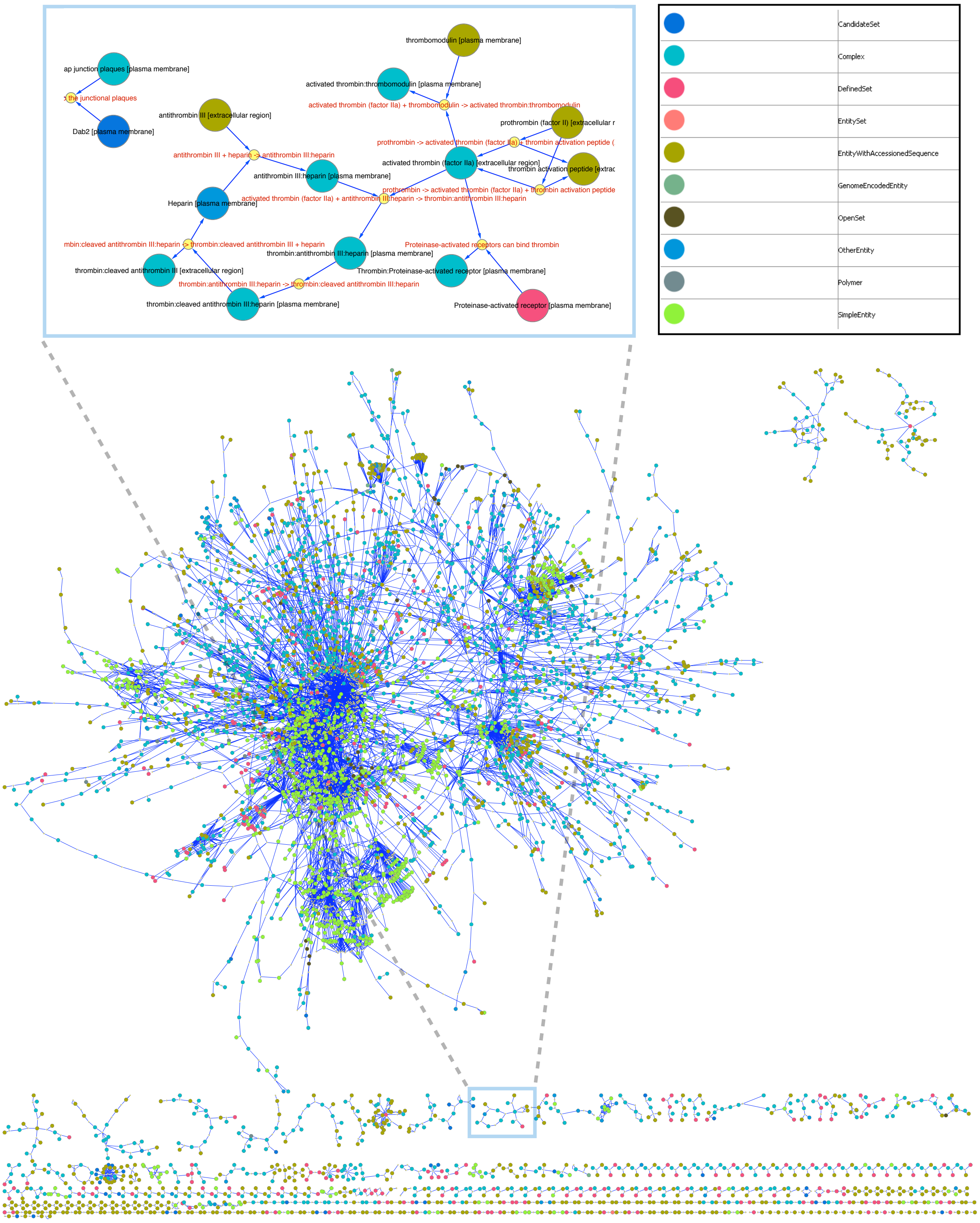

Figure: *Network generated with two SQL statements from a local instance of the Reactome database (version reactome_28), representing a bipartite graph of directed relationships between molecules (large nodes, colored according to legend) and reactions (small yellow nodes). The first two queries (see below, query 1 & 2) followed by union merge are used to generate a network consisting of relationships between "molecules" and "reactions". To demonstrate the simplicity of using CytoSQL to enrich an existing network, useful node attributes wered added for reactions and molecules, with one extra statement (query 3). It loads molecule and reaction descriptions for selected (or all) nodes.*

Query 1: Loading Input - Reaction network

```
SELECT
      ReactionlikeEvent_2_input.input AS input,
      ReactionlikeEvent_2_input.DB_ID AS reaction,
      ReactionlikeEvent_2_input.input_class AS molecule_class,
      speciesinfo.eventID,
      speciesinfo.species,
      speciesinfo.speciesname,
      "reaction"
FROM
      ReactionlikeEvent_2_input
INNER JOIN
      (SELECT
            Event_2_species.DB_ID as eventID,
            species, _displayName AS speciesname
      FROM
            Event_2_species
      LEFT JOIN
            DatabaseObject
      ON
            Event_2_species.species = DatabaseObject.DB_ID)
      AS speciesinfo
ON
      ReactionlikeEvent_2_input.DB_ID = speciesinfo.eventID
WHERE
      speciesinfo.speciesname = "Homo sapiens"
```

CytoSQL Mode: Create Network
CytoSQL mappings: source node, target node, source, edge, source, source, target

(Note: the 7th "reaction" field in the SELECT statement is a fixed value column that is mapped as a target node attribute, to facilitate the differentiation of "reaction" nodes from "molecular" nodes.)

Query 2: Loading Output - Reaction network

```
SELECT
      ReactionlikeEvent_2_output.DB_ID AS reaction,
      ReactionlikeEvent_2_output.output AS output,
      ReactionlikeEvent_2_output.output_class AS molecule_class,
      speciesinfo.eventID,
      speciesinfo.species,
      speciesinfo.speciesname,
      "reaction"
FROM
      ReactionlikeEvent_2_output
INNER JOIN
      (SELECT
            Event_2_species.DB_ID as eventID,
            species, _displayName AS speciesname
      FROM
            Event_2_species
      LEFT JOIN
            DatabaseObject
      ON
            Event_2_species.species = DatabaseObject.DB_ID)
      AS speciesinfo
ON
      ReactionlikeEvent_2_output.DB_ID = speciesinfo.eventID
```

```
WHERE
      speciesinfo.speciesname = "Homo sapiens"
```

<u>CytoSQL Mode</u>: Create Network
<u>CytoSQL mappings</u>: source node, target node, target, edge, target, target, source

The networks created with Query 1 and Query 2 are merged with the "Union" option.

(Note: the 7th "reaction" field in the SELECT statement is a fixed value column that is mapped as a source node attribute, to facilitate the differentiation of "reaction" nodes from "molecular" nodes.)

Query 3: Loading node descriptions

```
SELECT
      DB_ID,
      _displayName
FROM
      DatabaseObject
WHERE
      DB_ID = ?
```

<u>CytoSQL Mode</u>: Update node attributes
<u>CytoSQL bind variable</u>: ID
