# Supplementary material for: Flexible network reconstruction from relational databases with Cytoscape and CytoSQL
Source: BMC Bioinformatics. 2010 Jul 1;11:360. doi: 10.1186/1471-2105-11-360 (PMC2910028; doi:10.1186/1471-2105-11-360)
Supplement: Additional file 3 — AdditionalFile3.pdf - Application case 3. A pdf document that lists the queries and shows the generated network of application case 3: Use of public, remote relational databases. [file 1471-2105-11-360-S3.PDF]

Application 3: BioWarehouse

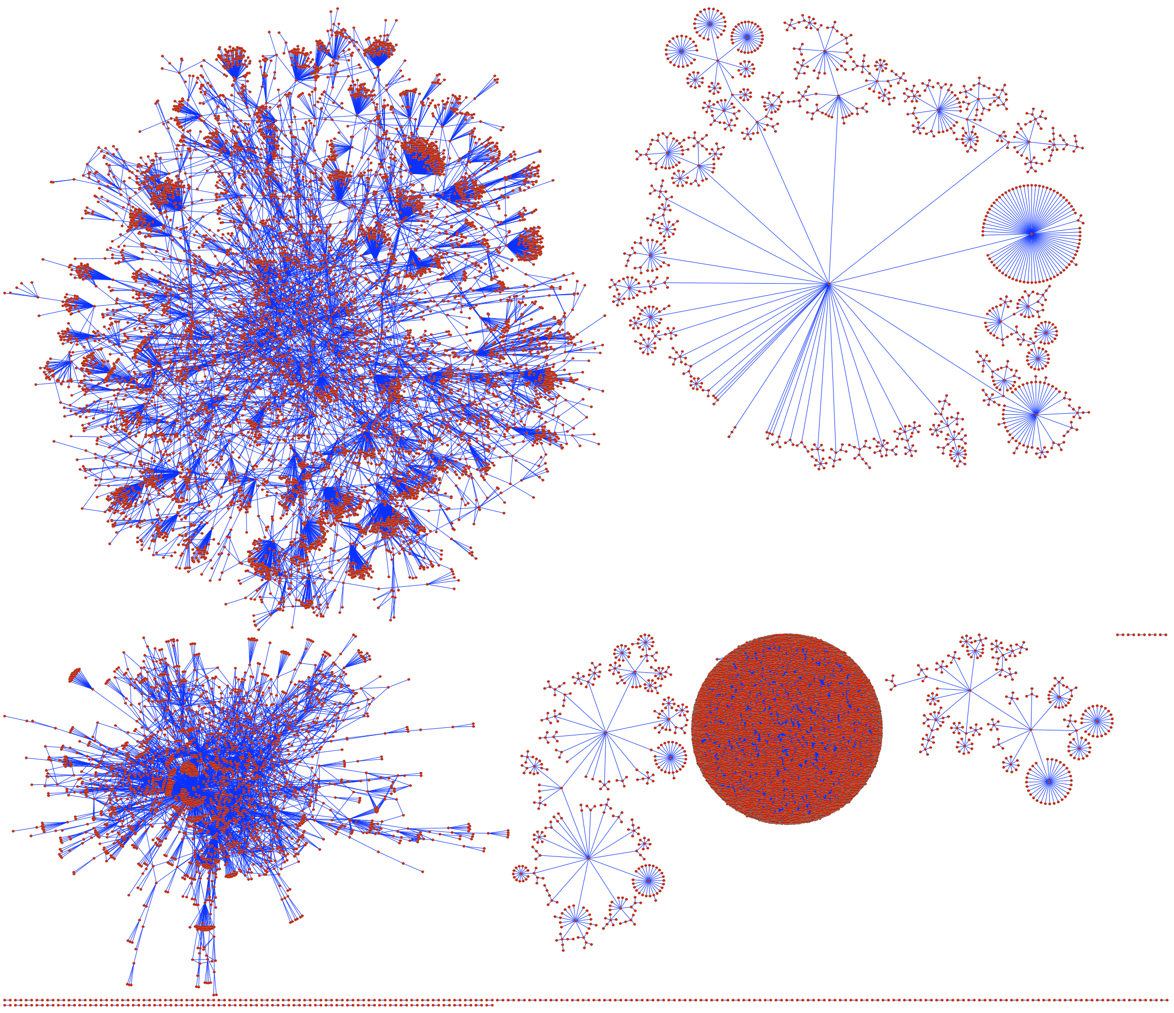

Figure: *Network visualisation of BioWarehouse Term relationships. A public instance of the 'BioWarehouse', a Bioinformatics toolkit to integrate biological data from multiple databases into a so-called "data warehouse" (available after free registration), was queried. With a single query, a network representation was constructed for the relationships between different "terms" in the warehouse. It reveals how terms belonging to different ontologies group together, and whether and how terms within an ontology are hierarchically organized.*

Query 1: Loading a term relationship network

```
SELECT
	TermRelationship.TermWID,
	TermRelationship.RelatedTermWID,
	TermRelationship.Relationship
FROM
	TermRelationship
```

CytoSQL Mode: Create Network
CytoSQL mappings : source node, target node, edge
